# Longitudinal Study of Adolescent Brain Connectivity Development Using Sign‐Aware Graph Theory Metrics

**DOI:** 10.1002/hbm.70549

**Published:** 2026-06-06

**Authors:** Subhasri Viswanathan, Sean Spinney, Jeremy J. Watts, Patricia Conrod

**Affiliations:** ^1^ Department of Neuroscience University of Montréal, CHU—Sainte Justine Azrieli Research Centre Montreal Quebec Canada; ^2^ CHU—Sainte Justine Azrieli Research Centre Montreal Quebec Canada; ^3^ Department of Psychiatry and Addiction University of Montreal, CHU—Sainte Justine Azrieli Research Centre Montreal Quebec Canada

**Keywords:** adolescent, brain development, graph theory, segregation and integration, sensorimotor—association axis

## Abstract

Adolescence is marked by significant changes in brain network organization that underlie cognitive and behavioral development. The sensorimotor‐association (SA) axis has been proposed as a hierarchical framework for understanding functional connectivity development, but most studies rely on cross‐sectional data and treat positive and negative connections equivalently. We analyzed longitudinal resting‐state fMRI data from 125 adolescents who passed quality control of a total of 151 (ages 12–18, 364 total scan sessions across three time points) using both functional connectivity strength and graph‐theoretical metrics, comparing results from absolute‐value networks (collapsing connection signs) versus sign‐aware approaches. Functional connectivity strength showed age‐related changes following the SA axis selectively for positive connections (*r* = −0.614, *p* < 0.001), with stronger effects in sensorimotor regions, while negative connections showed no SA alignment (*r* = 0.031, *p* = 0.803). Critically, graph‐theoretical measures revealed opposing developmental gradients depending on network construction: clustering coefficient and local efficiency showed association‐dominant patterns in absolute‐value networks (*r* = 0.317, *p* < 0.001; *r* = 0.427, *p* = 0.001) but sensorimotor‐dominant patterns in positive‐only networks (*r* = −0.225, *p* < 0.001; *r* = −0.277, *p* < 0.001). Participation coefficient, an integration‐based measure, showed no significant SA association in either construction. These findings demonstrate that developmental inferences critically depend on how negative connections and network topology are treated, challenging the notion of a single organizational gradient and highlighting the necessity of sign‐aware graph‐theoretical approaches for understanding adolescent brain maturation.

## 1 Introduction

Adolescence is a critical period of brain maturation marked by widespread changes in functional network organization (Dahl et al. 2018). Studies examining within‐ and between‐network connectivity have reported inconsistent findings, with some showing increased connectivity in networks such as the default mode network (DMN) and frontoparietal network (FPN) (Fair et al. 2009; Supekar et al. 2009), while others report decreased between‐network connectivity, potentially reflecting functional refinement (Fair et al. 2007; Power et al. 2010). These discrepancies likely stem from methodological variation, including network definitions, motion correction, and the predominance of cross‐sectional designs (Stevens 2016).

### 1.1 The Problem of Averaging Connection Signs

A critical but often overlooked issue is the treatment of negative functional correlations also known as anticorrelations. Most developmental studies either discard negative connectivity or convert it to absolute values when computing summary metrics, potentially conflating distinct biological processes. Positive and negative connectivity may show distinct developmental trends, with opposite trajectories in network properties (Hassett et al. 2024). Failure to distinguish them may lead to conflicting results (Hassett et al. 2024; Zhan et al. 2017), as these connection types may reflect fundamentally different aspects of brain organization following distinct developmental trajectories.

### 1.2 Graph Theory as a Complementary Framework

To better capture network complexity, recent work has turned to graph theory, which allows for a more fine‐grained and topologically informed characterization of large scale brain networks. In graph‐theoretical models, brain regions are treated as nodes, and their functional connections (FCs) as edges, enabling the quantification of global and local properties of network organization. Two key concepts derived from this framework are segregation which is the extent to which nodes form locally clustered communities, and integration which is the capacity for efficient communication across distant nodes. These measures provide insight into how the brain supports both specialized processing and global coordination and are particularly well‐suited for tracking developmental reorganization (Bullmore and Sporns 2009, 2012). These measures have shown relationships with cognitive performance and can predict individual differences (Gu et al. 2015; Markett et al. 2018; Wu et al. 2013). However, most graph‐theoretical studies face the same methodological challenge: they typically use absolute‐value correlation matrices, treating positive and negative connections equivalently (Rubinov and Sporns 2011; Wang et al. 2011), introducing critical interpretive ambiguities. While recent work has begun addressing these concerns through signed graph approaches (Hassett et al. 2024; Rubinov and Sporns 2011), systematic investigation of how edge sign treatment alters developmental inferences remains limited.

### 1.3 The Biological Significance of Anticorrelations

Accumulating evidence suggests negative correlations reflect meaningful aspects of brain organization rather than mere noise. Task‐based fMRI studies demonstrate neural reciprocal inhibition between competing networks, with resting‐state anticorrelations matching regions showing task‐based mutual suppression (Jack et al. 2013). Computational modeling confirms that anticorrelations contain significant information improving model performance (Parente and Colosimo 2018). Developmentally, anticorrelations strengthen selectively with age, particularly between association regions (Chai et al. 2014), with recent work showing increases in negative edge weights concurrent with decreases in positive weights, interpreted as enhanced network segregation (Hassett et al. 2024). However, these studies have not systematically examined whether positive and negative connectivity develop along the same spatial gradients or follow distinct organizational principles.

### 1.4 The Sensorimotor‐Association (SA) Axis

An emerging framework for understanding brain development is the SA axis, describing a hierarchical gradient from primary sensorimotor to transmodal association cortices (Pines et al. 2022; Sydnor et al. 2021). This axis aligns with the principal gradient of FC (Margulies et al. 2016) and strengthens across adolescence (Dong et al. 2021). Recent work by Luo et al. (2024) reported that age‐related changes in FC strength align with the SA axis, with stronger effects in sensorimotor regions. However, by averaging positive and negative correlations into a single summary statistic, this approach may obscure critical aspects of network reorganization. Association regions exhibit extensive anticorrelations with other networks (Fox et al. 2005), and developmental changes in these anticorrelations may represent functional differentiation rather than reduced connectivity. Without distinguishing connection signs, it remains unclear whether reported SA gradients reflect true developmental timing differences or measurement artifacts.

We address these gaps by investigating age‐related changes in functional network organization during adolescence using a sign‐aware, graph‐theoretical approach. We leverage longitudinal resting‐state fMRI data spanning ages 12–18 to examine how network properties develop along the SA axis and how these patterns depend on the treatment of positive versus negative connections and based on topological graph theory measures.

Our approach involves two key strategies. First, we assess age‐related changes in FC strength, comparing traditional absolute‐value approaches with separate analysis of positive and negative connectivity to test whether these connection types show distinct spatial patterns and developmental trajectories. Second, we apply graph‐theoretical measures (local clustering coefficient, local efficiency, and participation coefficient) to quantify segregation and integration, comparing results from absolute‐value networks (standard approach) with positive‐only networks (preserving edge sign) to test whether methodological choices regarding anticorrelations alter conclusions about developmental change.

By integrating sign‐aware connectivity analysis with graph‐theoretical characterization, this study provides a more refined understanding of adolescent brain network development with important methodological implications, demonstrating that developmental trajectory conclusions depend critically on how negative correlations are treated in network analyses.

## 2 Methods

This study used data from the NeuroVenture Trial, a longitudinal neuroimaging add‐on to the larger Co‐Venture Trial, a school‐based longitudinal cohort study investigating substance use prevention and brain development in Canadian adolescents (O’Leary‐Barrett et al. 2017). NeuroVenture involved repeated MRI scanning and behavioral assessments at three time points: baseline (T1, mean age ≈13.7 years), a 2‐year follow‐up (T2, mean age ≈14.9), and a 4‐year follow‐up (T3, mean age ≈17.4). A total of 151 adolescents were recruited, with imaging data available for 150 participants at T1, 145 at T2, and 131 at T3. Refer Table 1 for the number of scans retained at each time point and mean frame wise displacement values.

**TABLE 1:** Demographic and motion characteristics across timepoints.

Time point	Total complete scans (*N*)	Scans retained after QC (FD < 0.5 mm)	Mean age (years)	Males	Females	Mean FD (mm)
T1	150	116	13.71	52	64	0.1354
T2	145	123	14.94	55	68	0.1325
T3	131	125	17.41	58	67	0.1215

Participants were excluded if they had a diagnosis of a major neurodevelopmental disorder (e.g., autism), uncorrectable visual or auditory impairments, severe psychiatric illness (e.g., schizophrenia, bipolar disorder), current use of central nervous system–acting medications, or contraindications for MRI (e.g., metallic implants, dental braces) (Bourque et al. 2016).

At each time point, substance use was assessed using the DEP‐ADO questionnaire (Landry et al. 2005), a validated screening tool designed to detect alcohol and drug use problems in adolescents. Alcohol and cannabis use scores were included as covariates in statistical models to control for potential confounding effects of substance use on brain development.

Resting‐state functional MRI data were acquired using a Siemens 3 T scanner with the following parameters: repetition time (TR) = 2300 ms, echo time (TE) = 30 ms, voxel size = 3.5 mm^3^, and 152 volumes per scan, resulting in a total scan duration of 5 min and 56 s. Data were acquired using an interleaved slice acquisition protocol. High‐resolution T1‐weighted anatomical images were also collected to support spatial normalization and cortical surface reconstruction.

All MRI data were preprocessed using the fMRIPrep v20.2.3 pipeline (Esteban et al. 2019), which includes skull stripping and anatomical segmentation using ANTs and FreeSurfer, slice‐timing correction, motion correction, spatial normalization to MNI152NLin2009cAsym space, and co‐registration of functional images to the corresponding T1‐weighted anatomical scans. Confound regression included nuisance regressors such as framewise displacement (FD), DVARS, and components derived from anatomical and temporal CompCor (Behzadi et al. 2007), along with temporal derivatives and high‐pass filtering. Functional data were resampled back to MNI space.

Further denoising steps were performed using Nilearn, including the removal of frames with FD > 0.5 mm or standardized DVARS > 1.5, linear detrending, and band‐pass filtering (0.01–0.1 Hz). Time series were extracted from 333 regions of interest (ROIs) defined by the Gordon atlas (Gordon et al. 2016). The z‐score normalized time series was then used to calculate Pearson correlation coefficients between all ROI pairs to generate FC matrices. Diagonal elements were removed prior to further analysis. A detailed description of the fMRIPrep preprocessing pipeline and the full confound regression strategy used for denoising is provided in Supporting Information.

### 2.1 Graph Construction and Thresholding

To quantify large‐scale network topology, we constructed undirected, weighted graphs from the z‐transformed Pearson correlation matrices. Modules were defined based on the network assignments from the Gordon Atlas (Gordon et al. 2016). Specifically, each of the 333 ROIs was assigned to one of 13 networks: Somato Motor hand, Somato Motor mouth, Visual, Auditory, Retrosplenial‐Temporal, Cingulo‐Parietal, Fronto‐Parietal, Cingulo‐Opercular, Ventral Attention, Salience, Dorsal‐Attention, Default, and None. This approach ensures consistency with the broader literature and allows interpretation relative to well‐established functional systems.

Throughout this manuscript, we distinguish between:

1. Functional networks: Canonical brain systems defined by the Gordon atlas.

(e.g., DMN, Salience Network, Visual Network)

2 Network construction methods: Approaches for building graphs from correlation matrices

Absolute‐value networks—|*r*| values, treating positive and negative edges equivalently.

Positive‐only networks—Only positive correlations retained, negative values set to zero.

Signed networks—Preserve edge polarity (not implemented due to sparsity after thresholding; see below).

3 Connection types: Properties of individual edges

Positive connections: *r* > 0 (functional synchrony).

Negative connections: *r* < 0 (anticorrelations).

### 2.2 Treatment of Edge Signs

We employed two approaches to handle positive and negative functional connections:

1. *Absolute‐value networks* (standard approach): All correlation values were converted to their absolute values prior to graph construction. This approach ensures that connectivity strength reflects the magnitude of functional coupling between regions regardless of sign, under the assumption that both positive and negative correlations can reflect meaningful communication patterns in developing brain networks (Rubinov and Sporns 2010; Wang et al. 2011).
2. *Positive‐only networks* (sign‐aware approach): To assess the impact of treating anticorrelations as equivalent to positive connections, we created parallel networks preserving only positive edges. Negative correlations were set to zero before applying proportional thresholding. This approach allowed us to directly compare graph‐theoretical measures derived from networks that include versus exclude anticorrelations.

Additionally, for FC strength analyses, we calculated the sum of all positive edges and the sum of all negative edges to each node separately, yielding positive‐only and negative‐only FC strength measures.

For graph construction, we applied proportional thresholding to retain the top 25% of edges by weight for all primary analyses. This approach ensures consistent graph density across subjects and time points, facilitating comparability of graph metrics (Garrison et al. 2015; Wijk et al. 2010). To reduce spurious short‐range correlations caused by signal leakage, edges connecting node pairs separated by less than 30 mm (geodesic distance) were excluded (Demeter et al. 2023).

Due to the relatively low proportion of negative edges (~16% of all connections), proportionally thresholded negative‐only networks were too sparse for reliable graph‐theoretical analysis. Therefore, signed analyses focused on comparing absolute‐value versus positive‐only network measures. A detailed justification of this approach and additional methodological considerations are provided in Supporting Information.

All graph measures were computed using the weighted, undirected version of the adjacency matrix via the Brain Connectivity Toolbox for Python (BCTpy).

To characterize segregation and integration, we calculated three node‐level graph measures:

#### 2.2.1 Local Clustering Coefficient

The local clustering coefficient $C_{i}$ quantifies the extent to which a node's neighbors are also connected, reflecting local modularity and segregation. For weighted, undirected networks, it is defined as (Fagiolo 2007; Onnela et al. 2005; Watts and Strogatz 1998):

$$C_{i}=\frac{1}{k_{i}\left(k_{i}-1\right)}\sum_{j,k}{\left(w_{\mathrm{ij}}w_{\mathrm{jk}}w_{\mathrm{ki}}\right)}^{1/3}$$

where $k_{i}$ is the degree of node *i*, and $w_{\mathrm{ij}}$ is the edge weight between nodes *i* and *j*. Higher $C_{i}$ values indicate stronger clustering among a node's neighbors.

#### 2.2.2 Local Efficiency

Local efficiency $E_{\mathrm{loc}}\left(i\right)$ captures the efficiency of information transfer between a node's neighbors when the node itself is removed, reflecting the resilience and redundancy of local subnetworks. It is defined as (Fagiolo 2007; Latora and Marchiori 2001; Onnela et al. 2005; Rubinov and Sporns 2010)

$$E_{\mathrm{loc}}\left(i\right)=\frac{1}{\left|N_{i}\right|\left(\left|N_{i}\right|-1\right)}\sum_{j\neq k\in N_{i}}\frac{1}{d_{\mathrm{jk}}}$$

where $N_{i}$ is the set of neighbors of node *i*, and $d_{\mathrm{jk}}$ is the shortest path between nodes *j* and *k* in the subgraph formed by $N_{i}$.

#### 2.2.3 Participation Coefficient

The participation coefficient $P_{i}$ measures how evenly a node distributes its connections across different functional modules, indexing global integration (Guimerà and Nunes Amaral 2005):

$$P_{i}=1-\sum_{m=1}^{M}{\left(\frac{k_{i}^{m}}{k_{i}}\right)}^{2}$$

where $k_{i}$ is the total strength of node *i*, $k_{i}^{m}$ is the strength of connections between node *i* and module m, and *M* is the number of modules. Nodes with high $P_{i}$ connect broadly across modules, while low $P_{i}$ values indicate within‐module specialization.

#### 2.2.4 Analysis Plan

To investigate age‐related changes in brain network organization, we used Generalized Additive Mixed Models (GAMMs) to model nonlinear developmental trajectories while accounting for repeated measures across participants. GAMMs offer flexibility in capturing age‐related changes without imposing strict linear assumptions, which is particularly important for modeling neurodevelopmental processes during adolescence (Sanders et al. 2023; van Duijvenvoorde et al. 2019).

For each node and graph‐theoretical metric with the absolute and sign aware approach (FC strength, clustering coefficient, local efficiency, participation coefficient), we fit the following model:

$$\begin{array}{c} {\text{Metric}}_{\mathrm{ij}}=\beta_{0}+s\left({\mathrm{Age}}_{\mathrm{ij}}\right)+\beta_{1}{\text{Gender}}_{i}+\beta_{2}{\text{Alcohol}}_{\mathrm{ij}}\\ \;+\beta_{3}{\text{Cannabis}}_{\mathrm{ij}}+\beta_{4}FD_{\mathrm{ij}}+u_{i}+\varepsilon_{\mathrm{ij}} \end{array}$$

where ${\text{Metric}}_{\mathrm{ij}}$ denotes the network metric for subject *i* at timepoint *j*; $s\;\left({\mathrm{Age}}_{\mathrm{ij}}\right)$ represents a penalized thin‐plate regression spline representing a nonlinear effect of age; $\beta_{1}$, $\beta_{2,}$ $\beta_{3},\beta_{4}$ are gender, alcohol use, cannabis use, and FD are fixed‐effect covariates respectively. $u_{i}∼N\left(0,\sigma_{u}^{2}\right)$ is a random intercept for subject. $\varepsilon_{\mathrm{ij}}∼N\left(0,\sigma^{2}\right)$ is the residual error.

To quantify the unique contribution of age, we fit a corresponding null model that excluded the age term while retaining all other covariates and random effects. Age was coded as a continuous variable calculated from a participant's date of birth to the date of the MRI visit (range: 12.0–18.5 years). The delta *R* ^2^ (Δ*R* ^2^) for each node was computed as the difference in adjusted *R* ^2^ between the full and null models, providing a measure of how much variance in each graph metric was explained specifically by age.

### 2.3 Delta *R* ^2^ Calculation

$$\Delta R^{2}=R^{2}\_\mathrm{adj}\left(\text{Full Model}\right)-R^{2}\_\mathrm{adj}\left(\text{Null Model}\right)$$

This represents the unique variance explained by the age smooth function after controlling for covariates and subject‐level baseline differences.

We then assessed whether these age‐related changes followed a spatially organized pattern by correlating node‐level Δ*R* ^2^ values with the SA axis ranks using Spearman's correlation. To evaluate statistical significance, we applied the spin permutation test (Váša et al. 2018), which generates spatially rotated versions of the SA axis that preserve hemispheric symmetry and anatomical contiguity. A *p* value was derived by comparing the observed correlation to this null distribution across 10,000 permutations.

## 3 Results

### 3.1 Overview of Analytical Strategy

We investigated age‐related changes in functional brain networks across adolescence using three complementary approaches. First, we replicated prior findings showing that FC strength development follows the SA axis. Second, we examined whether this pattern differs when positive and negative connections are analyzed separately. Third, we assessed whether graph‐theoretic measures of network segregation and integration show similar spatial organization, and critically, whether these patterns depend on the treatment of negative connections. For all analyses, age effects were quantified as the variance explained by age (Δ*R* ^2^) from GAMMs, controlling for sex, substance use, and head motion.

### 3.2 FC Strength Follows the SA Axis

**FIGURE 1:**
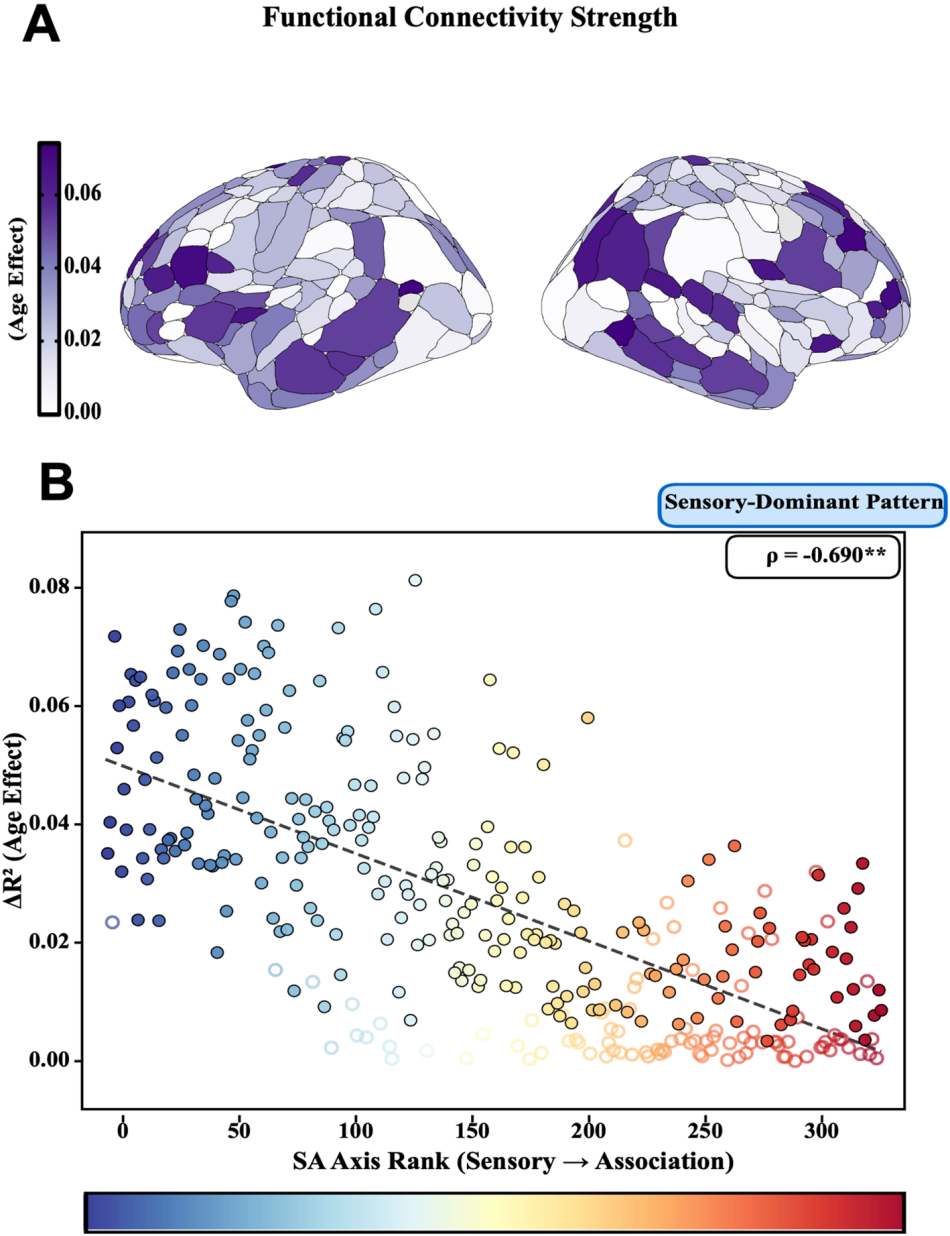
Age‐related changes in overall FC strength align with the SA axis. (A) Age‐related variance (Δ*R* ^2^) in functional connectivity strength projected onto cortical surface (Gordon atlas), with color intensity indicating magnitude of developmental change. (B) Correlation between Δ*R* ^2^ and SA axis rank (ρ = −0.69, *p* < 0.001, spin test), with ROIs color‐coded by SA rank (blue = sensorimotor, red = association). Filled circles: Significant ROIs (FDR *p* < 0.05); open circles: Nonsignificant.

We first confirmed that age‐related changes in overall FC strength follow a spatial gradient across the cortex. Age‐related variance (Δ*R* ^2^) in FC strength was strongest in primary sensorimotor regions and weakest in association cortices (Figure 1A). This pattern aligned systematically with the SA axis: regions with lower SA rank (sensorimotor cortex) showed substantially stronger age‐related changes than regions with higher SA rank (association cortex; ρ = −0.69, *p* < 0.001, spin test; Figure 1B). These findings replicate prior cross‐sectional observations (Luo et al. 2024) using longitudinal within‐person trajectories.

### 3.3 Overall Connectivity Patterns Show Distinct Distributions by Connection Polarity

**FIGURE 2:**
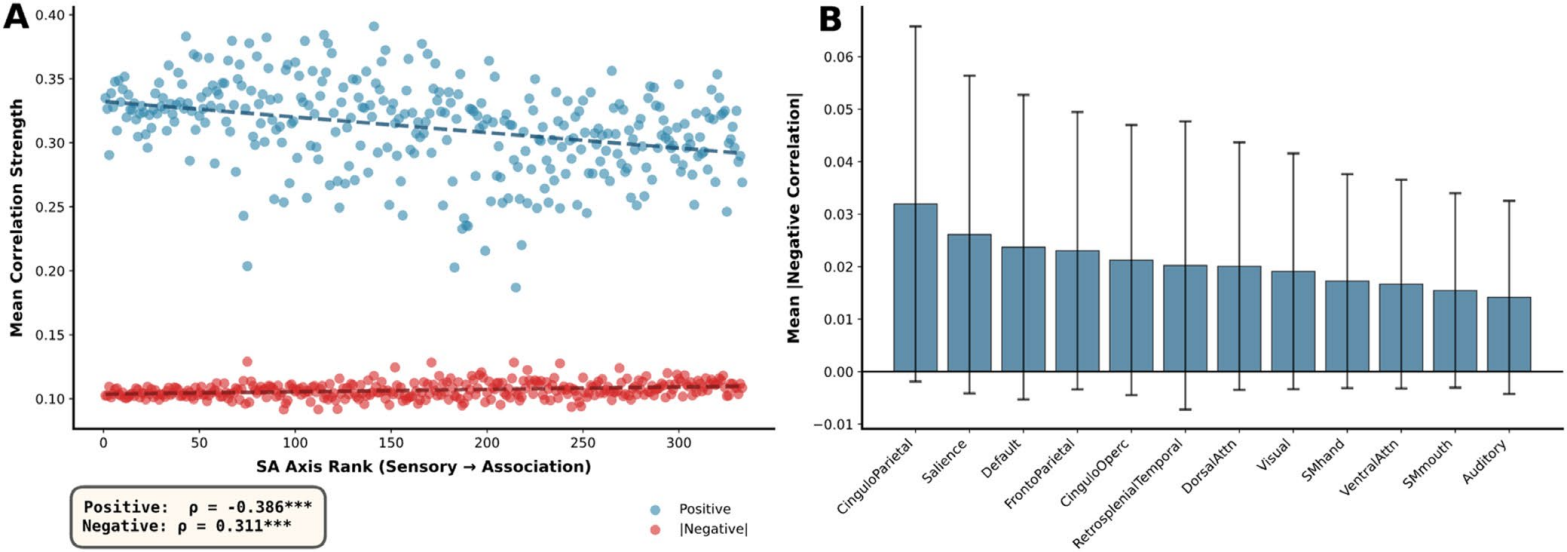
Overall connectivity strength differs between positive and negative connections. (A) Mean functional connectivity strength across SA axis for positive connections (blue) and absolute negative connections (red). (B) Mean absolute negative FC strength by functional network, showing stronger negative connectivity in association networks (Cingulo‐Parietal, Salience, Default) than sensorimotor networks (Visual, Somatomotor, Auditory). Association cortex exhibits more extensive anticorrelations than primary sensory regions. Bars represent the mean absolute negative connectivity within each functional network. Error bars indicate ±1 standard deviation across ROI‐level observations pooled over subjects and sessions.

Before examining developmental changes of positive and negative FC strengths, we characterized patterns of positive and negative connectivity across the cortical hierarchy. Mean positive FC strength showed a modest negative correlation with SA axis rank (Figure 2A, blue points), with sensorimotor regions exhibiting slightly stronger positive connections than association regions. In contrast, mean negative FC strength remained relatively constant across the SA axis, showing no systematic hierarchical organization (Figure 2A, orange points).

Network‐level analysis revealed that negative connections were most prevalent in association networks, particularly the Cingulo‐Parietal, Salience, and Default networks, while primary sensorimotor networks (Visual, Somatomotor, Auditory) showed weaker negative connectivity (Figure 2B). This distribution suggests that negative connections are concentrated in higher‐order association cortex, where they may support functional segregation through competitive or inhibitory dynamics.

### 3.4 Developmental Changes Follow the SA Axis Selectively for Positive Connections

**FIGURE 3:**
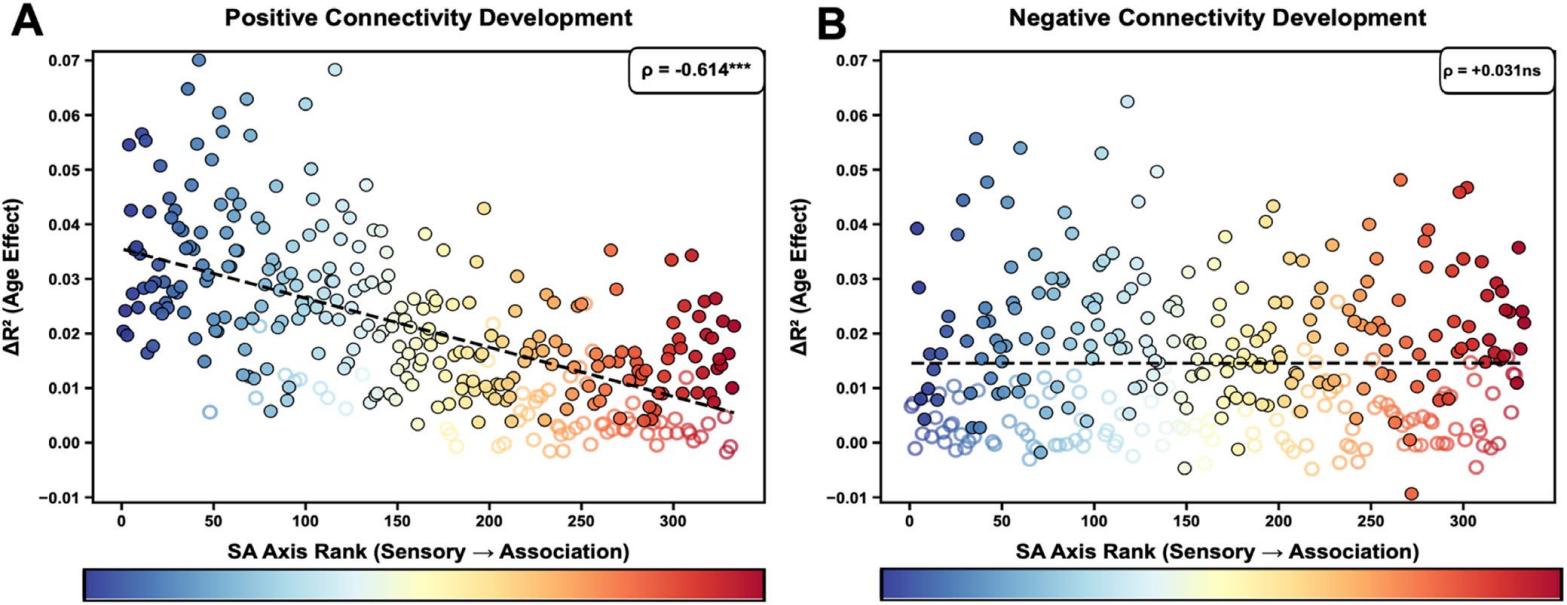
Developmental changes follow the SA axis selectively for positive connections. (A) Age‐related variance (Δ*R* ^2^) in positive connection strength shows strong negative correlation with SA axis rank (ρ = −0.614, *p* < 0.001, spin test), with sensorimotor regions (blue) exhibiting stronger developmental changes. (B) Age‐related variance in negative connection strength shows no relationship with SA axis rank (ρ = 0.031, *p* = 0.803, spin test), with uniform distribution across hierarchy. Filled circles: Significant (FDR *p* < 0.05); open circles: Nonsignificant; color gradient: Blue (sensorimotor) to red (association).

Having established sign differences, we examined whether age‐related changes in FC strength differed between positive and negative connections. Positive connections showed a strong negative correlation between Δ*R* ^2^ and SA axis rank (ρ = −0.614, *p* < 0.001; Figure 3A), replicating the pattern observed for overall FC strength: sensorimotor regions exhibited substantially stronger age‐related increases than association regions.

Strikingly, negative connections showed no significant relationship with the SA axis (ρ = 0.031, *p* = 0.803; Figure 3B), with age‐related variance distributed uniformly across the cortical hierarchy. This dissociation demonstrates that the SA gradient in FC development is driven specifically by positive functional connections, while negative connections mature according to distinct organizational principles independent of cortical hierarchy.

### 3.5 Graph‐Theoretic Measures Reveal Edge‐Sign Dependent Organization

Having established that positive and negative connections follow distinct developmental trajectories, we next examined whether graph‐theoretic measures of network organization also exhibit hierarchical patterns, and critically, whether these patterns depend on edge sign treatment.

### 3.6 Clustering Coefficient Shows Complete Reversal Depending on Edge Sign

**FIGURE 4:**
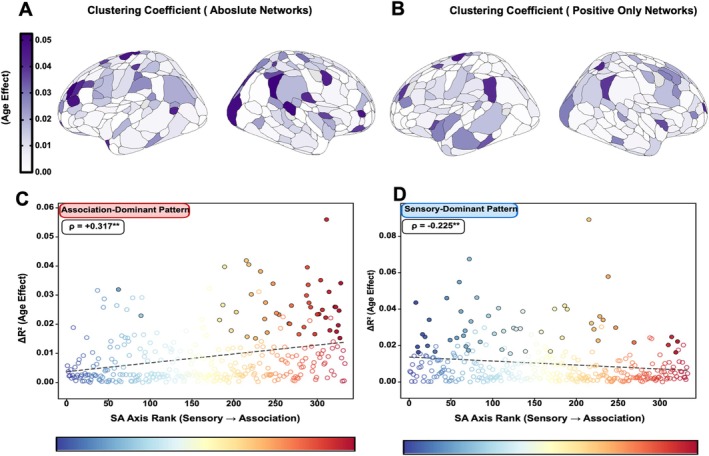
Clustering coefficient developmental gradient reverses with edge sign treatment. (A and B) Age‐related variance (Δ*R* ^2^) in clustering coefficient for absolute‐value networks shows association‐dominant pattern (ρ = +0.317, *p* < 0.001, spin test), with panel (A) showing cortical surface projection and panel (C) showing correlation with SA rank. (B–D) Same measure for positive‐only networks shows complete reversal to sensorimotor‐dominant pattern (ρ = −0.225, *p* < 0.001, spin test). ROIs color‐coded by SA rank (blue = sensorimotor, red = association); filled circles: Significant (FDR *p* < 0.05); open circles: Nonsignificant.

We first examined local clustering coefficient, which quantifies the extent to which a node's neighbors are interconnected. When computed on absolute‐value networks (treating positive and negative edges as equivalent positive weights), age‐related changes in clustering showed a positive correlation with SA axis rank (ρ = +0.317, *p* < 0.001; Figure 4A–C). This association‐dominant pattern indicated that higher‐order transmodal regions, particularly medial prefrontal and posterior parietal cortices, showed the strongest developmental increases in local clustering.

Remarkably, when the same analysis was performed on positive‐only networks (excluding negative connections), the spatial pattern completely reversed: age‐related changes in clustering showed a negative correlation with SA axis rank (ρ = −0.225, *p* < 0.001; Figure 4B–D). This sensorimotor‐dominant pattern revealed that primary sensorimotor regions exhibited the strongest developmental increases in clustering when negative connections were excluded.

This complete reversal demonstrates that negative connections fundamentally reshape the topology of developmental change. The association‐dominant pattern in unsigned networks reflects the influence of increasingly organized negative connectivity in higher‐order cortex, rather than genuine increases in positive local clustering.

### 3.7 Local Efficiency Confirms the Sensorimotor‐Dominant Pattern in Positive Networks

Local efficiency, another measure of network segregation reflecting the efficiency of information transfer among neighboring nodes, confirmed the sensorimotor‐dominant developmental pattern observed in positive‐only networks. Age‐related changes in local efficiency showed a negative correlation with SA axis rank (ρ = −0.277, *p* < 0.001). The consistency between clustering coefficient and local efficiency strengthens the conclusion that local network segregation in sensorimotor regions undergoes substantial developmental refinement during adolescence when negative connections are properly excluded from analysis. Refer Figure S3 for the brain plots and scatter plots.

### 3.8 Integration Measures Show No Systematic Hierarchical Organization

**FIGURE 5:**
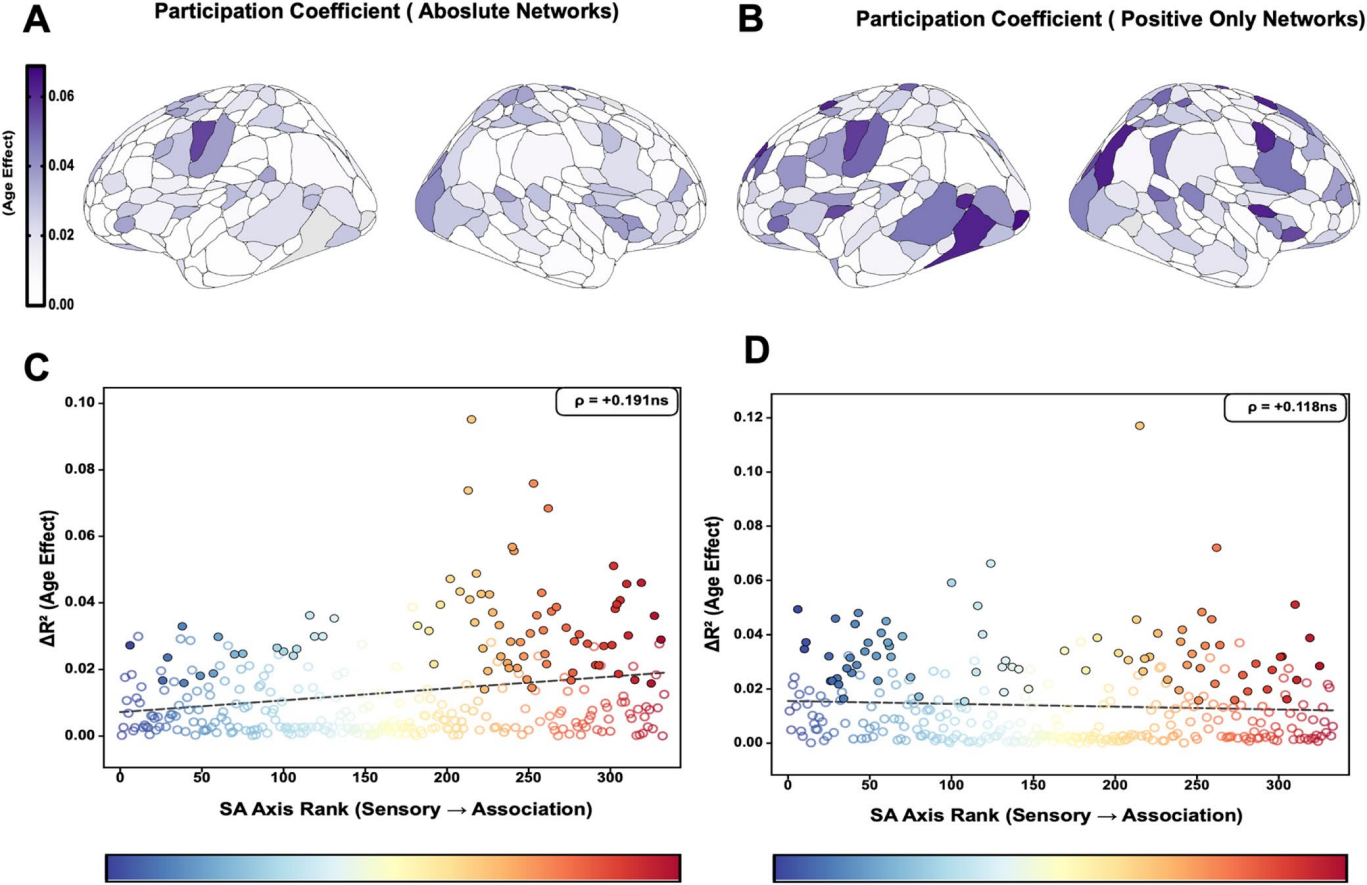
Integration measures show no SA axis organization. (A and B) Age‐related variance (Δ*R* ^2^) in participation coefficient projected onto cortical surface of absolute and positive only network, showing relatively uniform distribution without clear hierarchical pattern. (C and D) Correlation between Δ*R* ^2^ and SA axis rank shows no significant relationship (ρ = 0.191, *p* = 0.131, spin test). ROIs color‐coded by SA rank (blue = sensorimotor, red = association); filled circles: Significant (FDR *p* < 0.05); open circles: Nonsignificant.

In contrast to segregation measures, participation coefficient, which quantifies how evenly a node connects across different network communities, showed no significant relationship with the SA axis in either network construction. Age‐related changes in participation coefficient were not correlated with SA axis rank in absolute‐value networks (ρ = 0.191, *p* = 0.131) or in positive‐only networks (ρ = 0.118, *p* = 0.064; Figure 5). This null finding indicates that developmental changes in network integration are distributed relatively uniformly across the cortical hierarchy, following organizational principles distinct from those governing segregation or FC strength.

### 3.9 Segregation and Integration Develop in Partially Coordinated Fashion

To assess whether developmental changes in segregation and integration are coupled, we examined the correlation between Δ*R* ^2^ values for local clustering coefficient and participation coefficient across ROIs. A modest positive correlation was observed (ρ = 0.24, *p* < 0.01; Figure S4), indicating that regions showing strong developmental increases in segregation also tend to show increases in integration. This finding suggests that segregation and integration develop in a partially coordinated but not tightly coupled manner, supporting models of parallel refinement of specialized local circuits and distributed network hubs.

## 4 Discussion

This longitudinal study provides new insights into adolescent functional brain network development by demonstrating that the relationship between connectivity and cortical hierarchy depends critically on two analytical choices—whether positive and negative connections are examined separately, and whether connectivity is characterized by correlation strength or graph‐theoretic topology. Our findings reveal that the SA axis promoted as a universal developmental principle (Luo et al. 2024; Sydnor et al. 2023) applies selectively to positive connectivity and segregation based graph measures, while negative connections and integration‐based graph measures follow distinct organizational rules. These results underscore that a comprehensive understanding of brain maturation requires both signed network analysis and graph‐theoretic modeling.

### 4.1 Edge Sign Treatment Alters Developmental Inferences

Using longitudinal data spanning ages 12–18 with three timepoints per participant, we replicated the sensorimotor‐dominant developmental pattern for positive FC strength previously observed in large‐scale cross‐sectional studies (Luo et al. 2024). Critically, our within‐person trajectories confirm this pattern reflects true maturational change rather than cohort effects. However, negative FC showed no systematic relationship with cortical hierarchy, a dissociation invisible in absolute‐value analyses. This demonstrates that connection polarity is a first‐order property of network development and not a statistical nuisance (Fox et al. 2009; Murphy et al. 2009).

Separating positive and negative connections alone, however, proves insufficient. When we applied graph‐theoretic measures, specifically local clustering coefficient and local efficiency (Rubinov and Sporns 2010; Watts and Strogatz 1998), we observed a striking reversal in developmental gradients. Positive‐only networks showed stronger age‐related increases in segregation in sensorimotor regions, whereas absolute‐value networks (including negative edges) showed stronger changes in association regions. This complete inversion reveals that negative connections do not merely dilute the positive signal but actively reshape topological organization during adolescence. Understanding developmental network reorganization therefore requires both attention to connection sign and a topological framework capable of capturing higher‐order structure.

### 4.2 Revisiting the SA Axis as a Developmental Principle

Our replication of the sensorimotor‐dominant pattern for positive FC strength validates recent cross‐sectional findings (Luo et al. 2024) while extending them to within‐person developmental trajectories. Yet our data reveal a crucial boundary condition: SA‐axis alignment applies specifically to positive connectivity. Negative FC exhibited spatially uniform age‐related changes, suggesting that anticorrelated interactions mature according to principles distinct from the hierarchical gradient governing positive connections.

This finding extends previous observations that positive and negative connections evolve differently with age (Hassett et al. 2024). While Hassett et al. reported that positive connections become more efficient with small‐world properties across adolescence and negative connections emphasize functional differentiation, our longitudinal results add a spatial dimension: positive connections concentrate developmental change in sensorimotor cortex, whereas negative connections distribute change more evenly across cortical regions. Such uniformity suggests that negative connections may subserve domain‐general mechanisms maintaining segregation or competitive dynamics among large‐scale systems (Grayson and Fair 2017; Uddin 2015), maturing more synchronously across the cortex than their positive counterparts.

The neurophysiological interpretation of negative FC remains debated. Although early work attributed anticorrelations to preprocessing artifacts like global‐signal regression (Murphy et al. 2009), subsequent studies confirmed their presence in unregressed data and linked them to genuine inhibitory or competitive network dynamics (Fox et al. 2009; Chai et al. 2012). Our findings contribute a developmental perspective that negative connections display widespread but spatially nonspecific age‐related change, consistent with their proposed role in maintaining functional system balance and segregation rather than driving hierarchical specialization.

The graph‐theoretic reversal we observed, wherein segregation measures show opposite spatial patterns depending on edge sign treatment has important mechanistic implications. When negative edges are included in graph calculations, clustering coefficient and local efficiency show greater age‐related increases in association cortex. When negative edges are excluded, these same measures show greater increases in sensorimotor regions. This complete inversion underscores the interpretive risks of unsigned analyses.

In regions like the default‐mode and FPNs, where anticorrelations are strongest (Fox et al. 2005; Fransson et al. 2011), negative connections inflate apparent clustering when treated as positive weights. As these anticorrelations strengthen or reorganize with age (Marek et al. 2015; Sherman et al. 2014), they generate the appearance of association‐dominant increases in segregation. This observation clarifies why prior studies using absolute‐value networks often reported association‐driven maturation of modularity or segregation (Cao et al. 2016; Hwang et al. 2010). Those effects likely reflect developmental reorganization of negative connectivity rather than genuine increases in positive local clustering. For developmental inference, graph measures should therefore treat sign explicitly or use algorithms tailored for signed networks (Rubinov and Sporns 2011; Traag and Bruggeman 2009).

### 4.3 Multiple Organizational Gradients Guide Brain Development

The lack of SA alignment in negative connections and in integration measures like participation coefficient indicates that multiple organizational gradients govern adolescent brain development. Positive connectivity and segregation measures conform to a sensory‐to‐association trajectory, whereas integration and negative connectivity follow more distributed or orthogonal patterns (Margulies et al. 2016; Dong et al. 2021). This multidimensional view aligns with emerging frameworks describing several interacting gradients of cortical organization (Byeon et al. 2026). Recent work by Byeon et al. demonstrated that brain development involves coordinated maturation along at least three distinct axes namely sensory‐association (S‐A), task‐positive to default network (TP‐D), and somatomotor‐visual (SM‐V), each showing unique developmental trajectories and cognitive correlates. Similarly, (Bolt et al. 2022) used complex principal component analysis to decompose rs‐fMRI dynamics as traveling and standing waves along multiple cortical gradients, while (Xia et al. 2022) confirmed longitudinal strengthening of the unimodal‐to‐transmodal gradient originally described by (Margulies et al. 2016) alongside development along the task‐positive to default mode axis.

Within this framework, the SA axis remains a key descriptor of hierarchical processing from sensation to abstraction (Huntenburg et al. 2018; Mesulam 1998), but it does not capture all aspects of developmental reorganization. Negative connections may align more closely with gradients reflecting functional opposition (e.g., DMN–FPN anticorrelation; Fox et al. 2005), while integration metrics may follow gradients shaped by metabolic cost or communication efficiency (Bullmore and Sporns 2012; Tomasi et al. 2013). Recognizing this multiplicity of organizational axes provides a richer account of developmental plasticity than models anchored to a single hierarchical principle.

### 4.4 Coordinated But Distinct Development of Segregation and Integration

Our findings indicate that segregation and integration processes, though modestly correlated (ρ ≈0.24), develop partly independently. Local clustering and efficiency increased along hierarchical gradients, while the participation coefficient showed no significant SA alignment for either sign of connectivity. This supports models proposing parallel refinement of specialized local circuits and distributed network hubs (Baum et al. 2017; Fair, Cohen, Power, et al. 2009; Satterthwaite et al. 2013). Our longitudinal design confirms that both processes unfold within individuals over time, revealing coordinated yet distinct trajectories of functional specialization and integration.

### 4.5 Methodological and Theoretical Implications

These results have direct implications for developmental network neuroscience. Despite growing awareness that absolute‐value connectivity matrices conflate functionally distinct positive and negative relationships and distort graph‐theoretic topology (Hassett et al. 2024), they remain common in developmental graph analyses (Hallquist and Hillary 2018). Treating positive and negative connections equivalently transforms the topology of association cortex and can invert observed spatial gradients. We recommend that developmental analyses incorporate polarity explicitly, either by analyzing positive and negative subgraphs separately or by adopting graph algorithms designed for signed networks.

More broadly, our findings challenge the assertion that a single gradient universally governs brain development. Instead, adolescent maturation involves the interplay of multiple gradients reflecting different biological and computational demands. Positive connections strengthen hierarchically along the sensory–association continuum, negative connections may reinforce large‐scale segregation across the cortex, and integration measures reflect distributed hub formation unconstrained by cortical hierarchy.

### 4.6 Limitations and Future Directions

Several limitations merit acknowledgment. First, our analysis focused on a single cortical gradient (the SA axis) and a limited set of graph measures. Future work should examine how connectivity development relates to other proposed gradients and employ a broader range of network measures, including community detection, hub identification, and measures of network controllability. Second, while our longitudinal design with three timepoints is a strength over cross‐sectional studies, it limits fine‐grained characterization of developmental trajectories. Dense longitudinal sampling would enable more detailed characterization of developmental dynamics and identification of potential periods of accelerated change.

Additionally, we did not collect measures of pubertal development, which limits our ability to disentangle biological maturation from chronological age effects. Given the strong relationship between pubertal maturation and brain development during adolescence, future work should include pubertal staging (e.g., Tanner staging, hormonal assays) alongside chronological age. Recent work has demonstrated that pubertal stage can account for variance in FC beyond chronological age, particularly in limbic‐prefrontal circuits and DMN connectivity (Vijayakumar et al. 2018). The relative contributions of age‐dependent and puberty‐dependent processes to the network reorganization patterns we observe remains an important question for future research.

Third, the interpretation of negative connectivity remains contested, and different preprocessing pipelines (particularly global signal regression) can influence the presence and strength of negative connections. While we followed standard preprocessing protocols and did not include global signal regression in our models, our findings are unlikely to be simple artifacts of global signal regression, given that negative connections show systematic patterns and developmental changes, future work should examine the sensitivity of these results to preprocessing choices. Fourth, we focused on whole‐brain connectivity matrices without detailed examination of specific networks or connections. Targeted analyses of network pairs (e.g., default mode to frontoparietal) might reveal more granular insights into how negative connectivity develops.

The present study used the Gordon atlas (333 cortical regions) to define functional network parcellations, a widely used resting‐state–derived atlas that provides well‐characterized community structure and facilitates comparability across studies (Gordon et al. 2016). However, network communities were defined using adult resting‐state data, representing a potential limitation given that functional network organization continues to mature during adolescence. Future studies would benefit from parcellations derived from adolescent samples, such as those developed using large developmental cohorts, for example, (Hermosillo et al. 2024), to better capture age‐appropriate network architecture.

Despite these limitations, our findings demonstrate that understanding brain network development requires moving beyond averaged FC strength and acknowledging the fundamental heterogeneity of connections. Both the sign of connections and their topological organization matter, and these features follow distinct developmental principles not captured by a single hierarchical gradient.

## 5 Conclusions

This longitudinal study demonstrates that the relationship between FC development and cortical hierarchy is fundamentally contingent on analytical approach. Positive connections follow the sensorimotor‐to‐association gradient described in prior cross‐sectional work, which we replicate using within‐person trajectories. However, negative connections do not follow this pattern, showing spatially uniform developmental change. Graph‐theoretic measures of segregation show opposing patterns depending on whether negative connections are included in network construction, with signed analyses revealing a sensorimotor‐dominant gradient and unsigned analyses showing an association‐dominant gradient. Integration measures follow neither pattern, suggesting alternative organizational principles.

These findings challenge the notion that the SA axis is a universal organizing principle of brain development and reveal that negative connections play an active role in shaping developmental trajectories. Critically, our results demonstrate that neither signed network analysis nor graph theory alone would have been sufficient to reveal these patterns and that both are required. Future developmental neuroscience should adopt analytical frameworks that respect the heterogeneity of brain connections and employ graph‐theoretic tools designed for signed networks. Only through such approaches can we build accurate models of how brain architecture reorganizes during adolescence to support the emergence of mature cognitive abilities.

## Funding

This work was supported by operating grants from the Canadian Institutes of Health Research (CIHR) awarded to Patricia Conrod (FRN 170130 and FRN 126053). It was also supported by the CIHR grant awarded to Jeremy J Watts (FRN187907) and a Fonds de recherche du Quebec ‐ Sante (FRQS) grant (35450).

## Conflicts of Interest

The authors declare no conflicts of interest.

## Supporting information


**Table S1:** Generalized additive model (GAM) results for each node.
**Table S2:** Correlation with Sa axis for each graph measure after permutation.
**Figure S1:** Distribution of correlation values before and after Fisher z transform.
**Table S3:** Correlation between edges which were z score transformed vs the absolute.
**Figure S2:** Correlation of graph measures across multiple threshold densities compared with the test density (25%) used in the main analysis.
**Figure S3:** Local efficiency developmental gradient reverses with edge sign treatment.
**Figure S4:** Scatterplot of delta *R* ^2^ values for LCC versus participation coefficient. A positive trend is observed, with higher changes in segregation associated with higher changes in integration across ROIs.

## Data Availability

The data supporting the findings of this study are not publicly available due to the terms of the assent and consent forms. The data supporting the findings of this study are available upon reasonable request from Dr. Patricia Conrod.
