# Supplementary material for: Longitudinal Study of Adolescent Brain Connectivity Development Using Sign‐Aware Graph Theory Metrics
Source: Hum Brain Mapp. 2026 Jun 6;47(8):e70549. doi: 10.1002/hbm.70549 (PMC13241828; doi:10.1002/hbm.70549)
Supplement: Supplementary file 1 — Table S1: Generalized additive model (GAM) results for each node. Table S2: Correlation with Sa axis for each graph measure after permutation. Figure S1: Distribution of correlation values before and after Fisher z transform. Table S3: Correlation between edges which were z score transformed vs the absolute. Figure S2: Correlation of graph measures across multiple threshold densities compared with the test density (25%) used in the main analysis. Figure S3: Local efficiency developmental gradient reverses with edge sign treatment. Figure S4: Scatterplot of delta R 2 values for LCC versus participation coefficient. A positive trend is observed, with higher changes in segregation associated with higher changes in integration across ROIs. [file HBM-47-e70549-s001.docx]

**Supplementary Materials**

Dataset Description

The Neuroventure data is a subsample of 120 youth at high risk for substance misuse and 30 low-risk youth were recruited from the Co-Venture trial (Montreal, Canada) to take part in this 5-year follow-up neuroimaging study. The Co-Venture trial (Conrod et al, 2012) is a community-based cluster-randomized trial evaluating the effectiveness of school-based personality-targeted interventions on substance use and cognitive outcomes involving approximately 3800 Grade 7 youths. Cognitive tasks, structural and functional neuroimaging scans were conducted at baseline, and at 24- and 48-month follow-up.

Quality Control Description

All T1 images were visually quality checked for any brain abnormalities or other artifacts. CAT12 pipeline was ran to get Image Quality Reports, to select T1s with a higher score if there were more than 1 T1 available for a single session.

**fMRI Preprocessing**

Results included in this manuscript come from preprocessing performed using fMRIPrep 23.1.4 ([Esteban et al., 2019](https://doi.org/10.1038/s41592-018-0235-4); [Esteban et al., 2018](https://doi.org/10.5281/zenodo.852659); RRID:SCR_016216), which is based on Nipype 1.8.6 ([Gorgolewski et al., 2011](https://doi.org/10.3389/fninf.2011.00013); [Gorgolewski et al., 2018](https://doi.org/10.5281/zenodo.596855); RRID:SCR_002502).

Anatomical data preprocessing

All available T1-weighted (T1w) images were corrected for intensity non-uniformity (INU) using N4BiasFieldCorrection ([Tustison et al., 2010](https://doi.org/10.1109/TMI.2010.2046908)), distributed with ANTs ([Avants et al., 2008](https://doi.org/10.1016/j.media.2007.06.004); RRID:SCR_004757). Skull-stripping was performed with a Nipype implementation of antsBrainExtraction.sh, using the OASIS30ANTs template. Brain tissue segmentation into CSF, WM, and GM was performed with FAST (FSL; RRID:SCR_002823) [Zhang et al., 2001]. When multiple T1w images were available, they were robustly averaged using mri_robust_template ([Reuter et al., 2010](https://doi.org/10.1016/j.neuroimage.2010.10.004)). Cortical surface reconstruction was performed using FreeSurfer 7.3.2’s recon-all ([Dale et al., 1999](https://doi.org/10.1006/nimg.1998.0395); RRID:SCR_001847), and the brain mask was refined using Mindboggle-based reconciliation of ANTs- and FreeSurfer-derived segmentations ([Klein et al., 2017](https://doi.org/10.3389/fninf.2017.00027); RRID:SCR_002438). Spatial normalization to MNI152NLin2009cAsym space was performed via nonlinear registration with antsRegistration. Templates were accessed via TemplateFlow ([Ciric et al., 2022](https://doi.org/10.1038/s41597-022-01432-3); RRID:SCR_008796).

Functional data preprocessing

For each BOLD acquisition, a reference volume and skull-stripped version were generated. Head motion correction was performed using MCFLIRT ([Jenkinson et al., 2002](https://doi.org/10.1006/nimg.2002.1132)). Slice-timing correction was applied using AFNI’s 3dTshift ([Cox, 1996](https://doi.org/10.1006/cbmr.1996.0014); RRID:SCR_005927). BOLD data were resampled to native space for motion correction, and later co-registered to the T1w reference using FreeSurfer’s bbregister ([Greve and Fischl, 2009](https://doi.org/10.1016/j.neuroimage.2009.01.066)). Several confounding time-series were calculated, including framewise displacement (FD) (Power et al., 2014; Jenkinson et al., 2002), DVARS, and global signals from CSF, WM, and whole-brain masks. Physiological noise regressors were extracted using CompCor ([Behzadi et al., 2007](https://doi.org/10.1016/j.neuroimage.2007.04.042)), with both tCompCor and aCompCor components calculated.

Additional nuisance regressors included derivatives and quadratic terms of motion and global signals ([Satterthwaite et al., 2013](https://doi.org/10.1016/j.neuroimage.2012.11.049)), and outlier frames were flagged if FD > 0.5mm or DVARS > 1.5 SD. A PCA-based method for estimating noise from a cortical edge band was also used ([Patriat et al., 2017](https://doi.org/10.1016/j.neuroimage.2017.07.021)). All BOLD images were normalized to MNI152NLin2009cAsym using a single interpolation step with antsApplyTransforms (ANTs) and Lanczos interpolation ([Lanczos, 1964]), or mri_vol2surf for surface data.Many internal operations used Nilearn 0.10.1 ([Abraham et al., 2014](https://hal.inria.fr/hal-01123641); RRID:SCR_001362). For full pipeline details, refer to the [fMRIPrep documentation](https://fmriprep.org/en/stable/).

Resting-state fMRI data were denoised using a confound regression strategy implemented via nilearn.interfaces.fmriprep.load_confounds_strategy, which extracts nuisance regressors from fMRIPrep outputs. We applied the "high_pass", "motion", "wm_csf", and "scrub" strategies to address physiological noise, motion artifacts, and low-frequency drift. Specifically, the model included basic motion parameters (6 realignment regressors), average white matter (WM) and cerebrospinal fluid (CSF) signals, a high-pass filter with a cutoff of 0.01 Hz, and scrubbing of high-motion volumes defined as those with framewise displacement (FD > 0.5 mm) or standardized DVARS > 1.5. Scrubbed volumes were excluded and modeled with spike regressors. global signal regression was not included in our denoising strategy, in line with common practice for preserving individual differences in global network topology.

Following denoising, time series were extracted from 333 cortical parcels defined by the Gordon atlas using NiftiLabelsMasker, with spatial smoothing applied at 6 mm full-width at half maximum (FWHM), linear detrending, z-score standardization within each run, and temporal bandpass filtering between 0.01 and 0.1 Hz. All preprocessing and confound regression steps were applied in MNI152NLin2009cAsym space, consistent with outputs from fMRIPrep v20.2.3. This approach reduces non-neuronal signal variance and enhances the reliability of functional connectivity estimates for graph-theoretical analysis.

**Table S1. Generalized Additive Model (GAM) Results for Each Node**

This table added as an Excel file presents the results of the Generalized Additive Model (GAM) analysis across all nodes. The ΔR² values represent the variance explained by the age term, calculated as the difference between the full model (including age) and the null model (excluding age). Reported p-values are FDR-corrected to account for multiple comparisons. Significant nodes (p < 0.05, FDR-corrected) are indicated.

**Table S2 Correlation with Sa axis for each graph measure after permutation**

| Measure | Observed_Correlation | Permutation_P_Value |
| --- | --- | --- |
| Local_Clustering_Coefficient | 0.317055241519566 | 8E-04 |
| Local_Efficiency | 0.426663767872916 | 0.00 |
| Participation_Coefficient | 0.191147824832263 | 0.131 |

Additional Sensitivity analysis


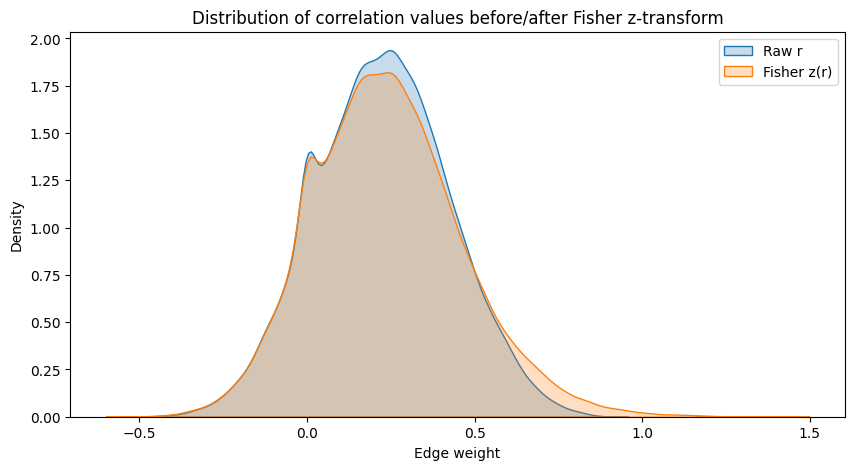


Fig S1 : Distribution of correlation values before and after Fisher z transform

**Table S3 : Correlation between edges which were z score transformed vs the absolute**

| Correlation Type | Correlation value |
| --- | --- |
| Pearson correlation ( r vs Z) | 0.9954 |
| Spearman Rank correlation ( r vs Z) | 1.0000 |


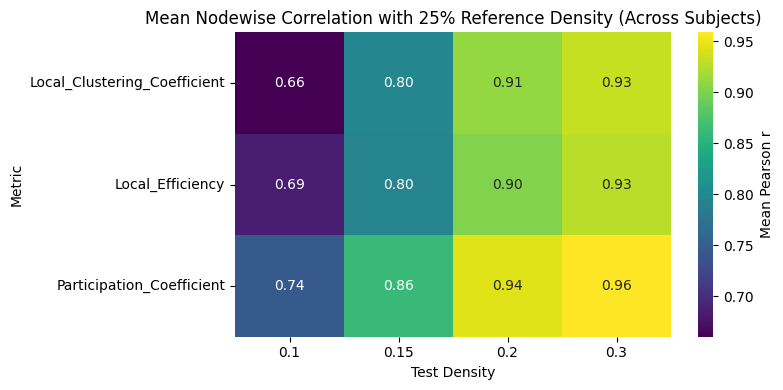


***Figure S2:*** *Correlation of graph measures across multiple threshold densities compared with the test density ( 25 % ) used in the main analysis.*


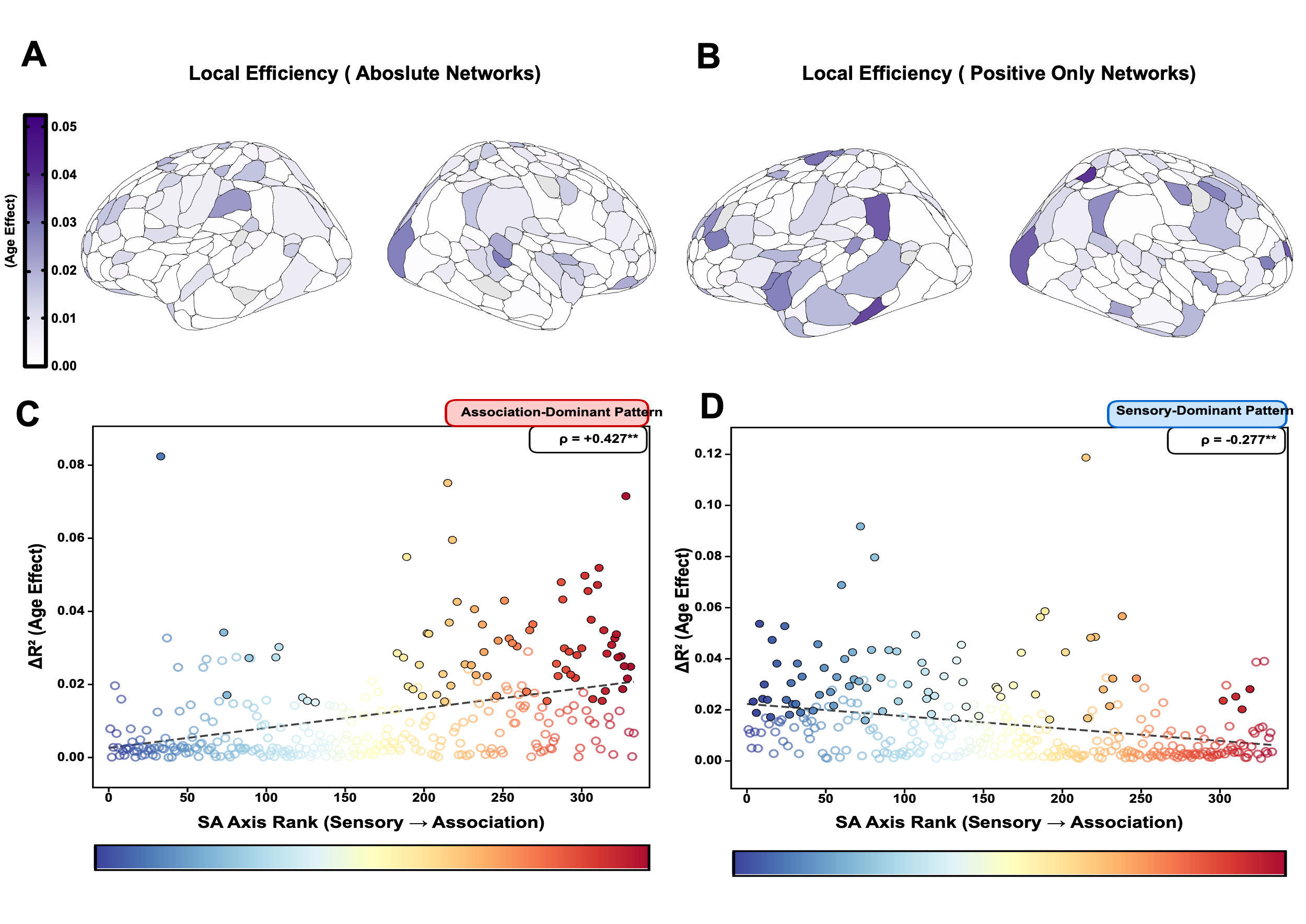


***Fig S3 Local Efficiency*** ***Developmental Gradient Reverses with Edge Sign Treatment***

*A-C) Age-related variance (ΔR²) in Local Efficiency for absolute-value networks shows association-dominant pattern (ρ = +0.317, p < 0.001, spin test), with panel A showing cortical surface projection and panel C showing correlation with SA rank. B-D) Same measure for positive-only networks shows complete reversal to sensorimotor-dominant pattern (ρ = -0.225, p < 0.001, spin test). ROIs color-coded by SA rank (blue = sensorimotor, red = association); filled circles: significant (FDR p < 0.05); open circles: non-significant.*


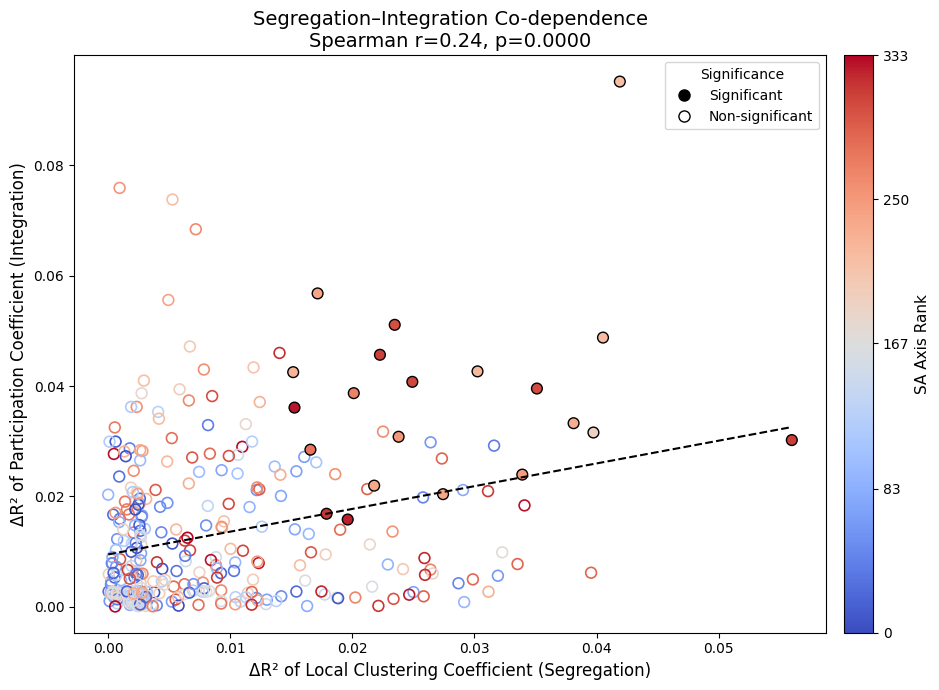


**Figure S4**: Scatterplot of delta R² values for LCC versus participation coefficient. A positive trend is observed, with higher changes in segregation associated with higher changes in integration across ROIs.
